# Gastroesophageal reflux disease and risk for arrhythmias: a Mendelian randomization analysis

**DOI:** 10.3389/fcvm.2024.1411784

**Published:** 2024-07-29

**Authors:** JunHao Liang, LuYi Tang, JinHui Yang, Yi Li, XiQiao Yang, ChiJun Hou

**Affiliations:** ^1^ Cardiology, Dongguan Hospital of Guangzhou University of Traditional Chinese Medicine, Dongguan, Guangdong, China; ^2^ Cardiology, Qidong City People’s Hospital, Nantong, Jiangsu, China

**Keywords:** gastroesophageal reflux disease, Mendelian randomization, paroxysmal tachycardia, arrhythmia, GWAS

## Abstract

**Background:**

Clinical observations and epidemiological studies suggest a potential linkage between gastroesophageal reflux disease (GERD) and arrhythmias, yet the underlying mechanism remains elusive. This study investigates the causal relationship between GERD and four types of arrhythmia through a genetic lens, employing Mendelian randomization analysis to elucidate the directionality of these associations.

**Methods:**

Selected single nucleotide polymorphisms (SNPs) from genome-wide association study (GWAS) data were utilized as instrumental variables. The inverse variance weighting (IVW) method, MR-Egger regression analysis, and the weighted median method were employed in two-sample Mendelian randomization analysis. Horizontal pleiotropy was detected and corrected using the MR-PRESSO test and MR-Egger regression. The stability and reliability of the Mendelian randomization results were assessed using the leave-one-out method, Cochran's Q test, and funnel plots. The causal relationship between GERD and four types of arrhythmias was evaluated using the odds ratio (OR).

**Results:**

IVW results indicated that GERD could increase the risk of arrhythmias. A one standard deviation increases in the logarithmically transformed GERD score resulted in a 34% increase in the risk of arrhythmia (OR = 1.34; 95% CI 1.19–1.51; *p* = 1.66E-06). No significant correlation was found between GERD and other arrhythmias.

**Conclusion:**

A causal relationship exists between GERD and arrhythmias, suggesting that GERD increases the risk of developing these arrhythmias.

## Introduction

Arrhythmia, the most prevalent cardiovascular disorders, are primarily observed in pathological states, encompassing both cardiac and non-cardiac conditions. Non-cardiac triggers of arrhythmias include hyperthyroidism, anemia, infection, and gastroesophageal reflux disease (GERD). These conditions are categorized into various types, such as atrial fibrillation, atrial flutter, supraventricular tachycardia, ventricular fibrillation, ventricular flutter, and conduction blocks. Cardiac arrhythmias affect over 700,000 people in England, with the most prevalent type, atrial fibrillation (AF), affecting up to 1% of the population and accounting for 1% of the entire NHS budget (1, 2). In the United States, the projected increase in arrhythmia cases to 16 million by 2050 is expected to result in considerable morbidity and mortality (3). This highlights the urgent need for strategies to prevent arrhythmia.Several arrhythmias we included in this study: paroxysmal tachycardia (paroxysmal tachycardia is regarded by clinicians as vegetative dystonia with crises and centres of overexcitatiou in different departments of the conduction apparatus of the heart.) (4), right bundle branch block, left bundle branch block, conduction disorders (RBBB, LBBB and conduction disorders: A block in the bundle branch of the electrical conduction system of the heart) (5).

GERD is characterized by the backflow of stomach contents into the esophagus, leading to discomfort and potential complications. Common symptoms include heartburn, characterized by a burning sensation beneath the sternum that ascends from the upper abdomen to the neck. GERD is highly prevalent, affecting approximately 20% of adults in the Western world (6). Population-based studies indicate its widespread presence in communities, with prevalence rates ranging from 10% to 30% (7).

Numerous prior studies have demonstrated that risk factors associated with arrhythmia, such as hypertension, diabetes, smoking, and dyslipidemia, elevate the risk of arrhythmias (8). Consequently, addressing these risk factors can alleviate the economic burden on patients, reduce the incidence of arrhythmias, and assist clinicians in adopting innovative treatment approaches (9). Several studies suggest a potential link between gastroesophageal reflux and arrhythmias, although the underlying mechanisms, possibly related to vagal tone and parasympathetic reflexes, remain unclear (10–12). Roemheld, in the last century, identified a correlation between the upper digestive tract and cardiovascular diseases, coining the term “gastrocardiac syndrome”. He demonstrated that esophageal stimulation could induce arrhythmias (13). Over the years, the investigation into the causal relationship between digestive and cardiovascular diseases has intensified. An observational studies indicate that esophageal acid stimulation can initiate a cardiac autonomic reflex, leading to arrhythmias (14). Previous research reveals that sensory input from the gut or other internal organs can influence heart function. For instance, rat studies have shown that bloating can affect cardiovascular regulation through the activation of neuronal circuits of excitation and inhibition (15). However, in humans, the effects of gastrointestinal stimulation on cardiac autonomic nervous function remain elusive. Tougas and colleagues investigated the impact of esophageal stimulation on cardiac autonomic function, applying mechanical or electrical stimulation to the esophagus and observing its effect on heart rate, thereby testing the hypothesis of a vagal afferent response (16). In summary, a potential correlation exists between GERD and arrhythmias, and this paper aims to determine whether a causal relationship exists between them through rigorous scientific methods.

Mendelian randomization (MR) is an analytical method employed to assess the observed causal relationships between modifiable risk factors and clinically relevant outcomes, adhering to the Mendelian laws of genetics, which dictate the random distribution of allele genes. MR analysis leverages genetic variation linked to an exposure as an instrumental variable to deduce causality with the outcome. Should a causal link exist between the exposure and outcome, the instrumental variable associated with the exposure will proportionally influence the outcome, thereby substantially reducing confounding and reverse causation (17).The objective of Mendelian randomization is to evaluate causal hypotheses within non-experimental data (18). To estimate the causal relationships between GERD and arrhythmias, we utilized pooled level data from several large consortia in a two-sample MR (TSMR) study.

## Materials and methods

### Study design

**Figure 1:**
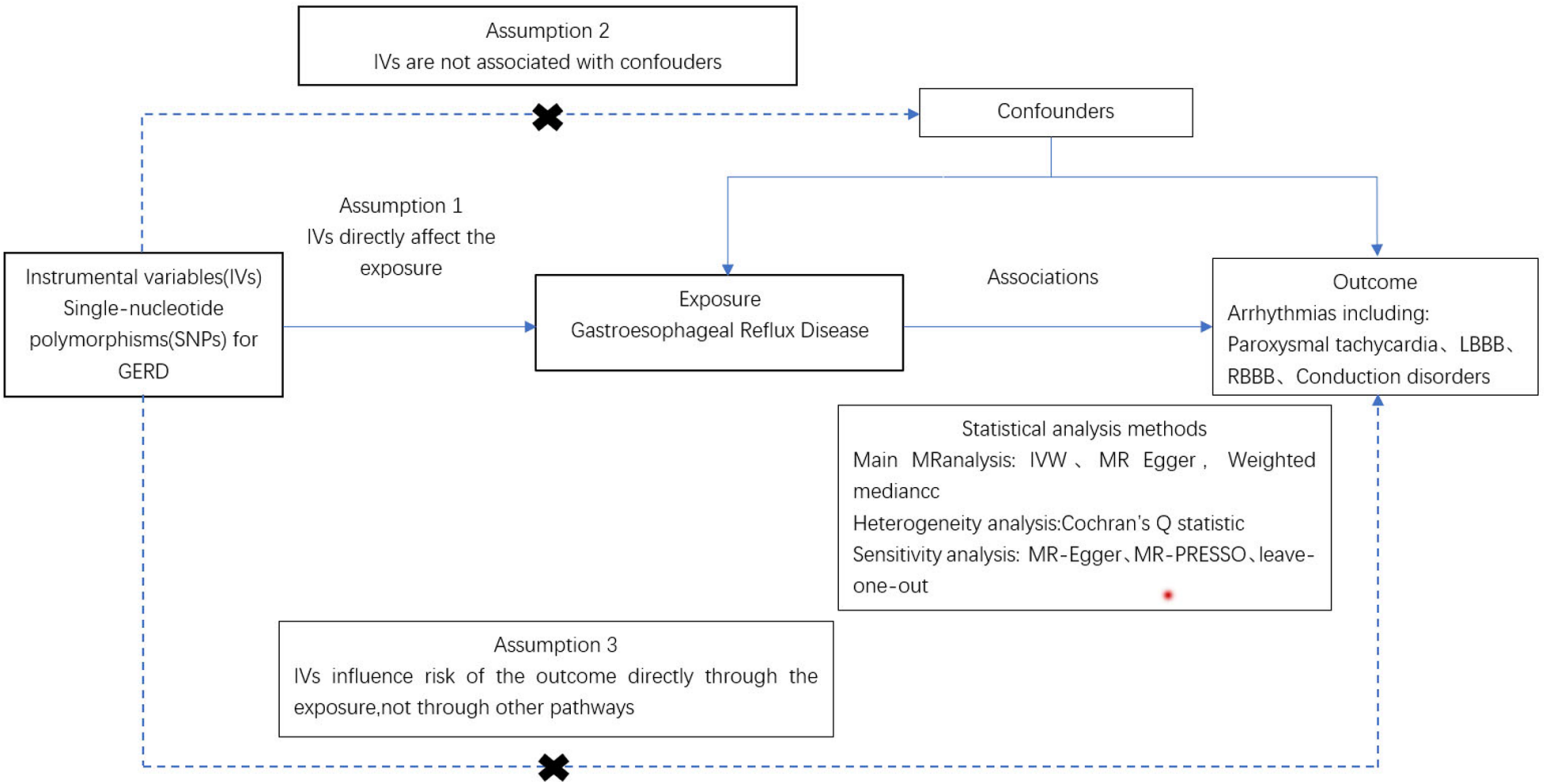
Procedure for an MR analysis of causal associations gastroesophageal reflux disease and risk for arrhythmia.

As illustrated in Figure 1, an MR study was designed to systematically explore the association between gastroesophageal reflux and arrhythmias risk. All studies included in our analysis received approval from their respective academic ethics review boards, with each participant providing written informed consent. The institutional review boards granted the necessary approvals. Notably, the current study, being a reanalysis of publicly available GWAS data, did not require additional ethical approval. This study adhered to the STROBE-MR (Strengthening the Reporting of Observational Studies in Epidemiology using Mendelian Randomization) guidelines (Supplementary Table S4).

### Data source

In this study, a comprehensive MR analysis was conducted to elucidate the causal relationship between GERD and arrhythmias (paroxysmal tachycardia, right bundle branch block, left bundle branch block, and conduction disorders), All the patients met the international diagnostic criteria for ICD. The GWAS data for GERD were sourced from the GWAS Catalog (ebi-a-GCST90000514), encompassing 129,080 cases and 473,524 controls of European ancestry (19),as the data related to GERD is derived from the GWAS database, the classification of GERD remains unknown to us. Genetic instrumental variables for the four arrhythmias were obtained from the R10 database of the FinnGen Biobank (20). Details are provided in Supplementary Table S1. To adhere to the two-sample MR study's assumption of independent samples for exposure and outcomes, we excluded GWAS pooled measurements of arrhythmias phenotypes with significant overlap with the GERD samples. The selected arrhythmias ensured all participants were of European descent, thereby reducing the risk of pleiotropic bias across the analyses (21). This study aimed to investigate the causal link between genetic predisposition to GERD and arrhythmias risk.

### Selection of instrumental variables (IVs)

MR analysis is predicated on three fundamental conditions: (1) the genetic variant is strongly associated with the exposure; (2) the genetic variant is not influenced by confounding factors; (3) the genetic variant is not affected by the outcome (21–23). Initially, a genome-wide significance threshold of 5 × 10^−8^ was applied to identify SNPs significantly associated with GERD (24). Secondly, to ensure the independence of SNPs, we required that those associated with exposure should not be in linkage disequilibrium (LD), setting the LD threshold at r^2^ = 0.001 distance greater than 10,000 KB (25). Furthermore, the F-statistic was calculated for the IVs to assess the presence of weak instrumental bias. The F-value was determined using the equation F = Beta2/Se2, where Beta represents the allele effect value, and Se represents the estimated standard error of Beta. To prevent any potential bias caused by weak IVs, only IVs with F > 10 were retained for further analysis (26). Lastly, SNPs meeting these criteria were verified using the Phenoscanner database (www.phenoscanner.medschl.cam.ac.uk) on March 10, 2024, to exclude any that might be associated with a confounding phenotype (27). SNPS not linked to phenotypes affecting the results were retained for further analysis.

### Mendelian randomization analysis

In this study, the inverse variance weighting (IVW) method served as the primary approach to estimate the causal effect. Within two-sample MR Analysis, IVW is regarded as a potent method for causality detection (28). Supplementary analyses were conducted using MR-Egger (29) and the weighted median methods (30). A series of sensitivity analyses were then performed. Initially, to mitigate the impact of heterogeneity on causal effect, Cochran's Q test was employed to assess heterogeneity. If *p* > 0.05, indicating that the influence of heterogeneity on the causal effect is negligible, the fixed effect model was applied. Conversely, the random effect model was utilized to minimize the impact of heterogeneity on the causal effect (Supplementary Table S5) (31). Subsequently, the MR-Egger intercept test was employed to detect horizontal pleiotropy (29). Additionally, the MR-PRESSO outlier test was utilized to identify and adjust for outliers among SNPs, thereby correcting for horizontal pleiotropy (32). To assess the robustness of the findings, a residual sensitivity analysis was conducted. The R programming language (version 4.3.1) facilitated the statistical analysis, employing the “TwoSampleMR” and “MRPRESSO” packages for MR analysis and the “forest plot” package for visualization. A *p*-value < 0.05 was deemed statistically significant.

## Results

### Instrumental variable selection

In this analysis, 79 SNPS associated with GERD and arrhythmias were identified. Allelic frequency palindromes within these SNPs were excluded, specifically rs2145318, rs2358016, rs9517313, and rs957345. The F statistic was calculated for each SNP, all demonstrating F values greater than 10, indicative of no weak instrumental bias. For detailed F values, refer to Supplementary Table S2. All selected SNPs were then verified using the Phenoscanner V2 database, resulting in no additional SNP exclusions. Ultimately, these 79 SNPs were employed as instrumental variables for GERD, with detailed information available in Supplementary Table.

### Two-sample Mendelian randomization analysis

**Figure 2:**
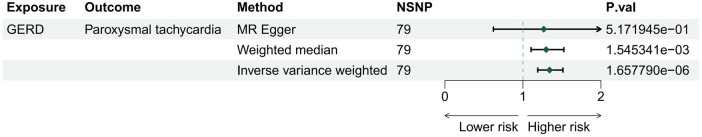
The risk association between gastroesophageal reflux disease and risk for arrhythmia in a forest plot.

Three methods (MR Egger, weighted median, and IVW) were employed to assess the causal relationship between GERD and arrhythmias. The IVW method, serving as the primary analytical approach, identified GERD as an independent risk factor for paroxysmal tachycardia (OR = 1.34; 95% CI 1.19–1.51; *p* = 1.66E-06), as depicted in Figure 2. Our analysis did not reveal significant causal associations between GERD and other arrhythmias, such as conduction disorders, right bundle branch block (RBBB), and left bundle branch block (LBBB), as detailed in Supplementary Table S3.

**Figure 3:**
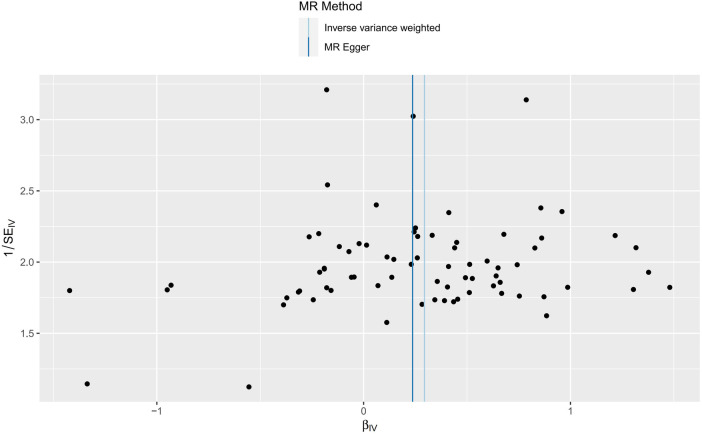
Scatter plot of MR analyses from gastroesophageal reflux disease and arrhythmia.

These findings indicate that GERD may elevate the risk of paroxysmal tachycardia, both epidemiologically and genetically, whereas no causal links were established between GERD and LBBB, RBBB, or conduction disorders. Scatter plots illustrating the estimated effect sizes for gene-predicted GERD and paroxysmal tachycardia are presented in Figure 3.

Furthermore, MR-Egger intercept and MR-PRESSO tests were conducted to detect pleiotropy, with *P*-values > 0.05 suggesting no pleiotropic interference. A leave-one-out analysis was also performed, sequentially excluding each SNP to verify the consistency of the causality results, which confirmed the robustness of the MR analysis findings (Supplementary Figure).

## Discussion

GERD, a prevalent condition of the digestive system, is notably linked to various cardiovascular diseases due to the unique anatomical proximity of the esophagus to the heart. Our MR analysis investigated the causal relationship between GERD and four arrhythmias, revealing a significant association between GERD and paroxysmal tachycardia. GERD could increase the risk of arrhythmias. A one standard deviation increases in the logarithmically transformed GERD score resulted in a 34% increase in the risk of arrhythmia. Several epidemiological studies have highlighted GERD's pivotal role in triggering and promoting arrhythmias. Huang et al. conducted a population-based study and demonstrated a higher incidence of future arrhythmias in patients with GERD compared to controls (HR of 1.31, 95% CI of 1.06–1.61, *P* = 0.013) as part of a nationwide cohort in Taiwan (33). Similarly, Kunz et al. reported the relative risk for developing arrhythmias in GERD patients compared with controls during healthcare encounters in the USA (HR of 1.39, 95% CI of 1.33-1.45) (34). However, it is important to note that this study did not observe an association between GERD and left bundle branch block(LBBB), right bundle branch block(RBBB), or conduction disorders. Despite some observational studies suggesting a link between GERD and arrhythmias (35), many predisposing factors for arrhythmias are similar to those associated with GERD, including increasing age, sleep apnea, obesity, and diabetes, making it challenging to distinguish GERD as a predisposing factor independent of common confounding factors The actual incidence of patients with both GERD and arrhythmias is reported to be 5% (36). Current studies, either too small or retrospective, fail to show a clear association between GERD and arrhythmias. In a meta-analysis involving 82 882 GERD patients and 29 671 patients without GERD, there was no statistically significant increase in the risk of arrhythmias (37). Therefore, we speculate that the lack of association between GERD and LBBB, RBBB, and conduction disorders may be related to the small size of study statistics or the presence of common confounding factors.

Clinical observational studies have consistently demonstrated a significant link between GERD and certain arrhythmias (35). Another research found a causal connection between gastroesophageal reflux and supraventricular tachycardia (38). Electrophysiological disturbances are crucial for the onset of paroxysmal tachycardia, with clinical evidence underscoring inflammation as a key factor in cardiac electrophysiological abnormalities (39). GERD triggers an increase in interleukin-8 mRNA (IL-8 mRNA) expression in esophageal mucosal cells, leading to neutrophil infiltration and an inflammatory response (40). This inflammation can cause myocardial damage and influence the electrophysiological remodeling of the myocardium by altering myocardial membrane potential, affecting cardiac ion channels, and impacting the action potential (39), ultimately resulting in paroxysmal tachycardia. In summary, we hypothesize that GERD, through local acidic irritation, prompts an inflammatory reaction in adjacent tissues, influencing systemic inflammation levels and, consequently, the heart's conduction system.

In addition, gastric reflux through esophageal acid stimulation can diminish coronary blood perfusion, subsequently elevating the risk of myocardial ischemia (41). We hypothesize that autonomic excitation is associated with sympathetic inhibition (42). Post-myocardial ischemia nerve growth is a significant arrhythmias contributor. Nerve growth factor, by binding to the tyrosine protein kinase A receptor on the superior cervical ganglion surface, activates the extracellular regulatory protein kinase (ERK1/2), catalyzes serine phosphorylation at S727 in STAT3, and activates STAT3 along with downstream signaling molecules to facilitate nerve growth (43). Tougas et al. observed that autonomic balance was disrupted following esophageal acid injection (44), suggesting that esophageal acid signals could influence cardiac electrical rhythm, potentially leading to arrhythmias. Additionally, according to one study: the autonomic nervous system directly impacts arrhythmia, where the vagus nerve and parasympathetic preganglionic fibers converge on the fat pad between the superior vena cava and the aorta to form a ganglionic plexus, thereby innervating the sinoatrial and atrioventricular nodes (45).

The strengths of our two-sample bidirectional MR study include the following: Firstly, the MR analysis method allows for the emulation of randomized controlled trials within an observational setting, providing higher-level experimental evidence through a cost-effective approach. Moreover, the MR methodology effectively circumvents reverse causation and confounding variables. Secondly, all instrumental variables (IVs) employed in the MR analysis were meticulously selected to ensure result accuracy. Thirdly, due to data being sourced from distinct tissues, there was minimal overlap between exposures and outcomes in the samples. Finally, our analysis's findings could have significant implications for healthcare policy, as elucidating a causal link between GERD and paroxysmal tachycardia may influence public health strategies concerning prevention and treatment.

Our study possesses several limitations. Firstly, the GWAS datasets utilized were exclusively derived from European populations, restricting the generalizability and applicability of our findings to diverse populations. Hence, further validation with a broader and more inclusive database is necessary. Secondly, the inherent constraints of the GWAS datasets necessitate additional basic research to substantiate causality and explore potential pathological mechanisms, pivotal for clinical interventions. Thirdly, the outcome data from GERD did not delineate the frequency and severity of GERD episodes, warranting further investigation into the relationship between GERD severity and the risk of paroxysmal tachycardia and other arrhythmias.

## Conclusion

In summary, our research underscores a potential causal link between GERD and arrhythmias. While the underlying mechanisms connecting GERD and arrhythmia remain elusive, GERD significantly contributes to arrhythmias risk assessment. Considering the causal association between GERD and arrhythmias, enhancing arrhythmias prevention strategies in individuals with GERD is advisable.

## Data Availability

The original contributions presented in the study are included in the article/Supplementary Material, further inquiries can be directed to the corresponding author.
