# Supplementary material for: Gastroesophageal reflux disease and risk for arrhythmias: a Mendelian randomization analysis
Source: Front Cardiovasc Med. 2024 Jul 29;11:1411784. doi: 10.3389/fcvm.2024.1411784 (PMC11317468; doi:10.3389/fcvm.2024.1411784)

Supplementary Material

**Supplementary Figure 1.** Leave-one-out plot and Funnel plot of MR analyses from Gastroesophageal reflux disease to Paroxysmal tachycardia

**
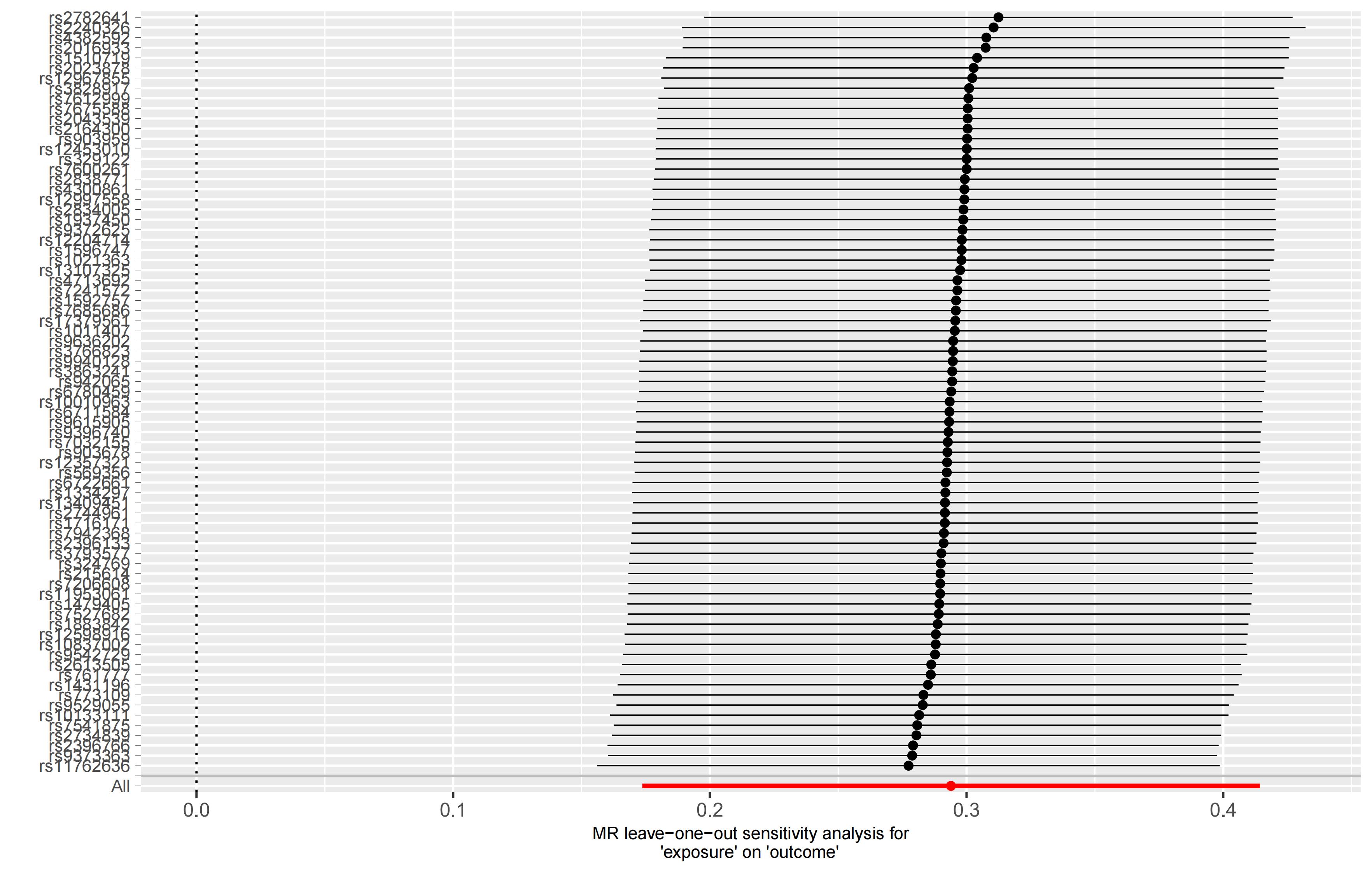
**

**
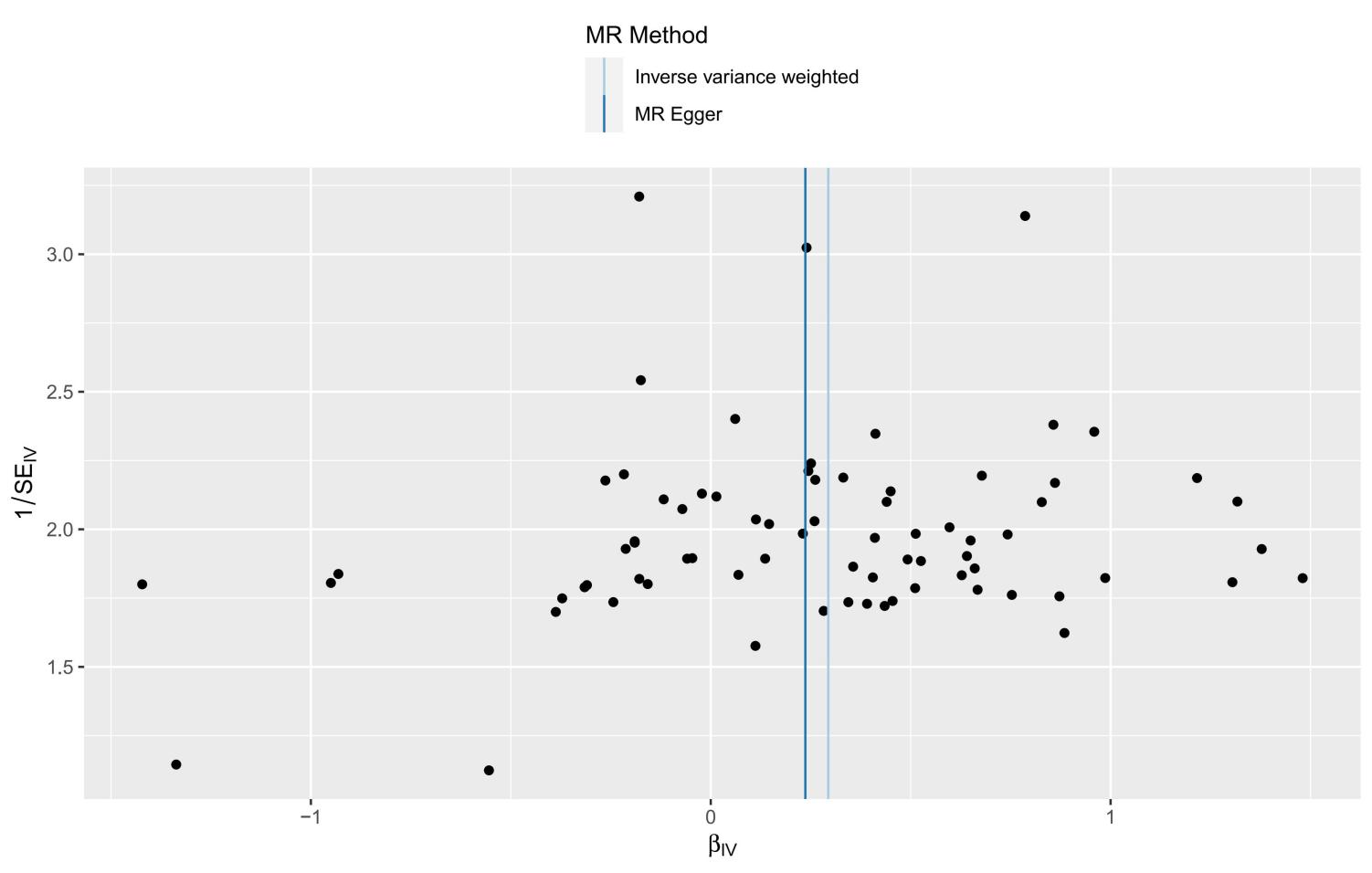
**

**Supplementary Figure 2.** Leave-one-out plot and Funnel plot of MR analyses from Gastroesophageal reflux disease to Right bundle branch block


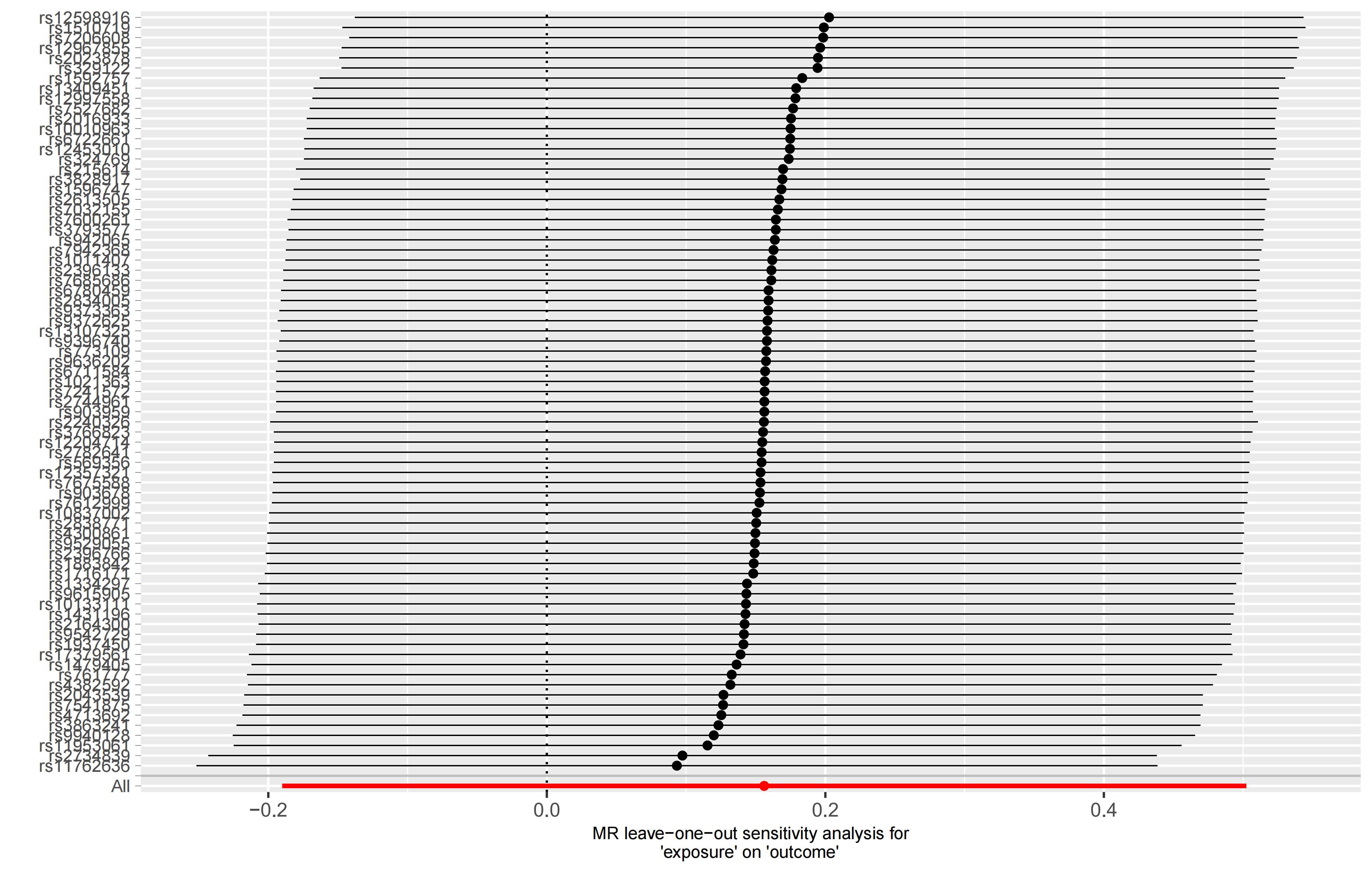


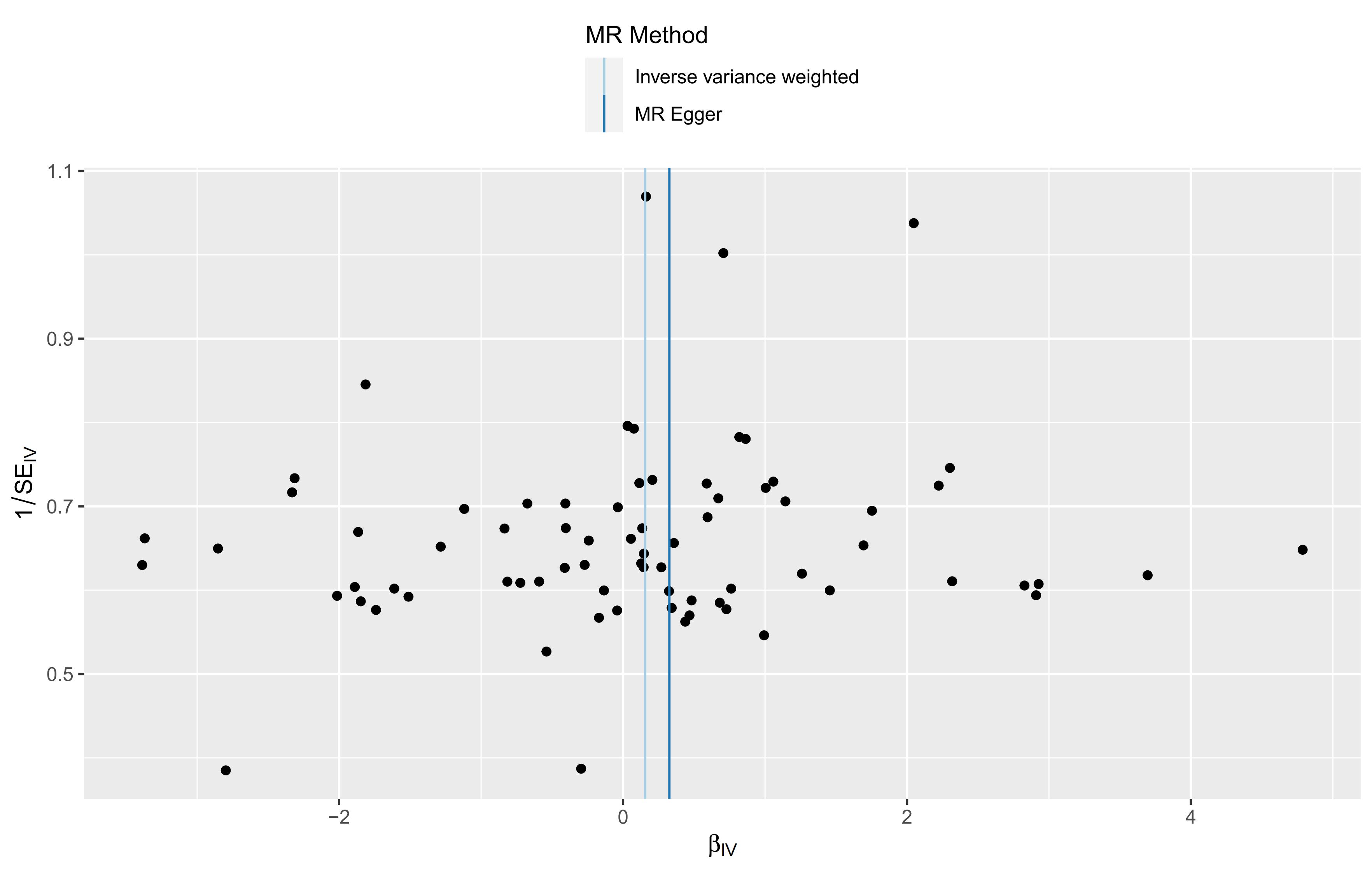


**Supplementary Figure 3.** Leave-one-out plot and Funnel plot of MR analyses from Gastroesophageal reflux disease to Left bundle branch block


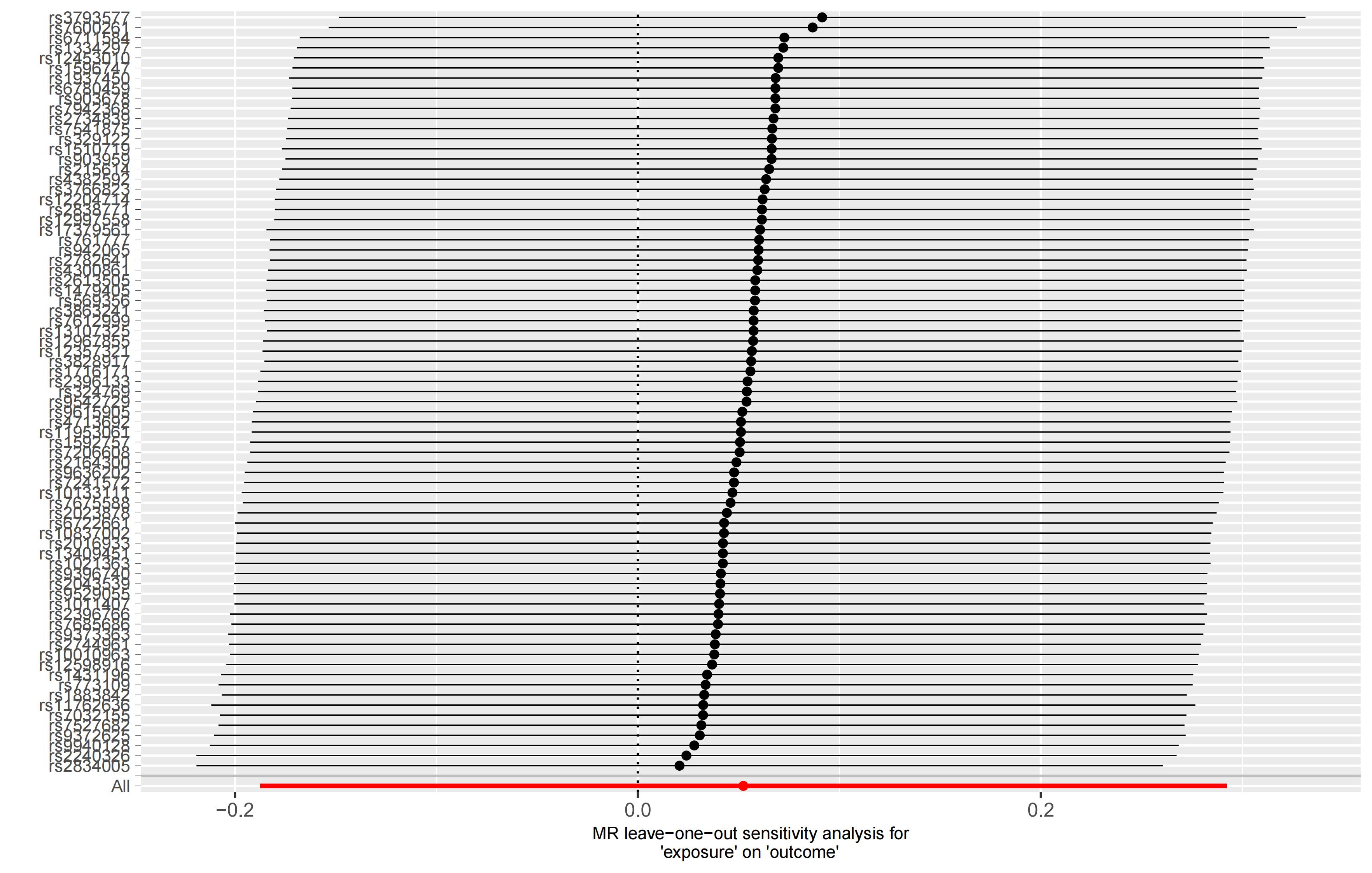


**
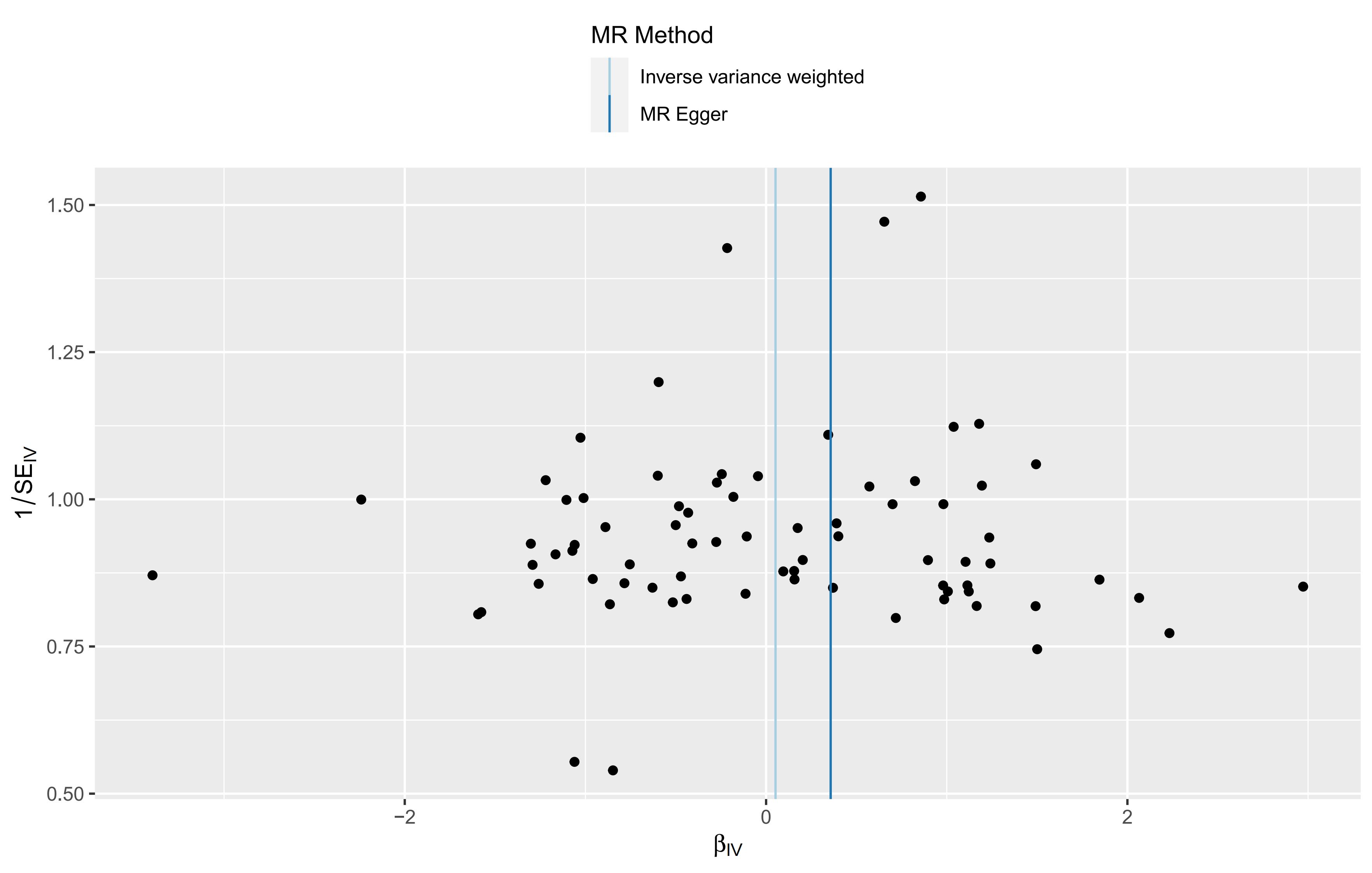
**

**Supplementary Figure 4.** Leave-one-out plot and Funnel plot of MR analyses from Gastroesophageal reflux disease to Conduction disorders

**
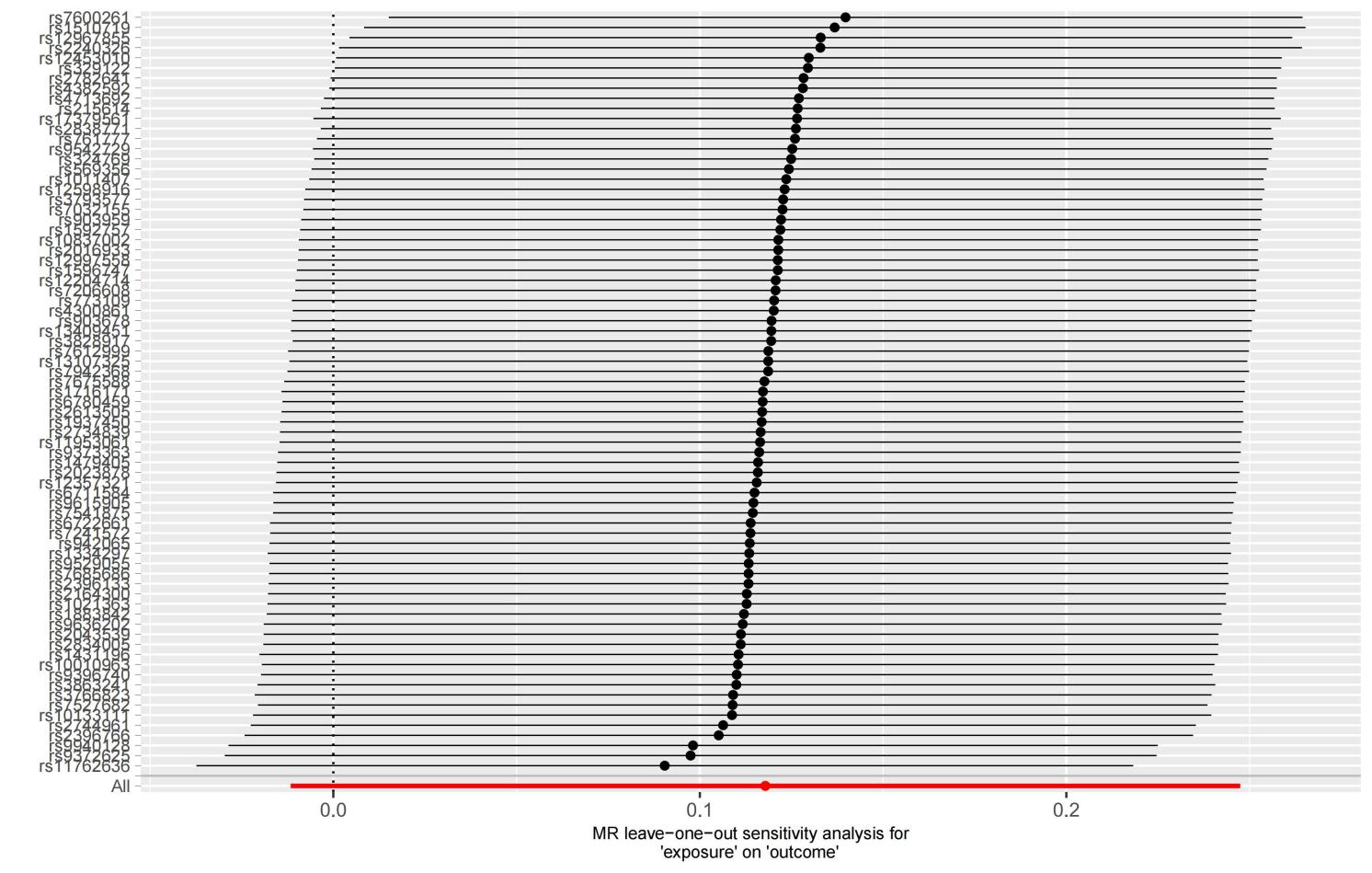
**


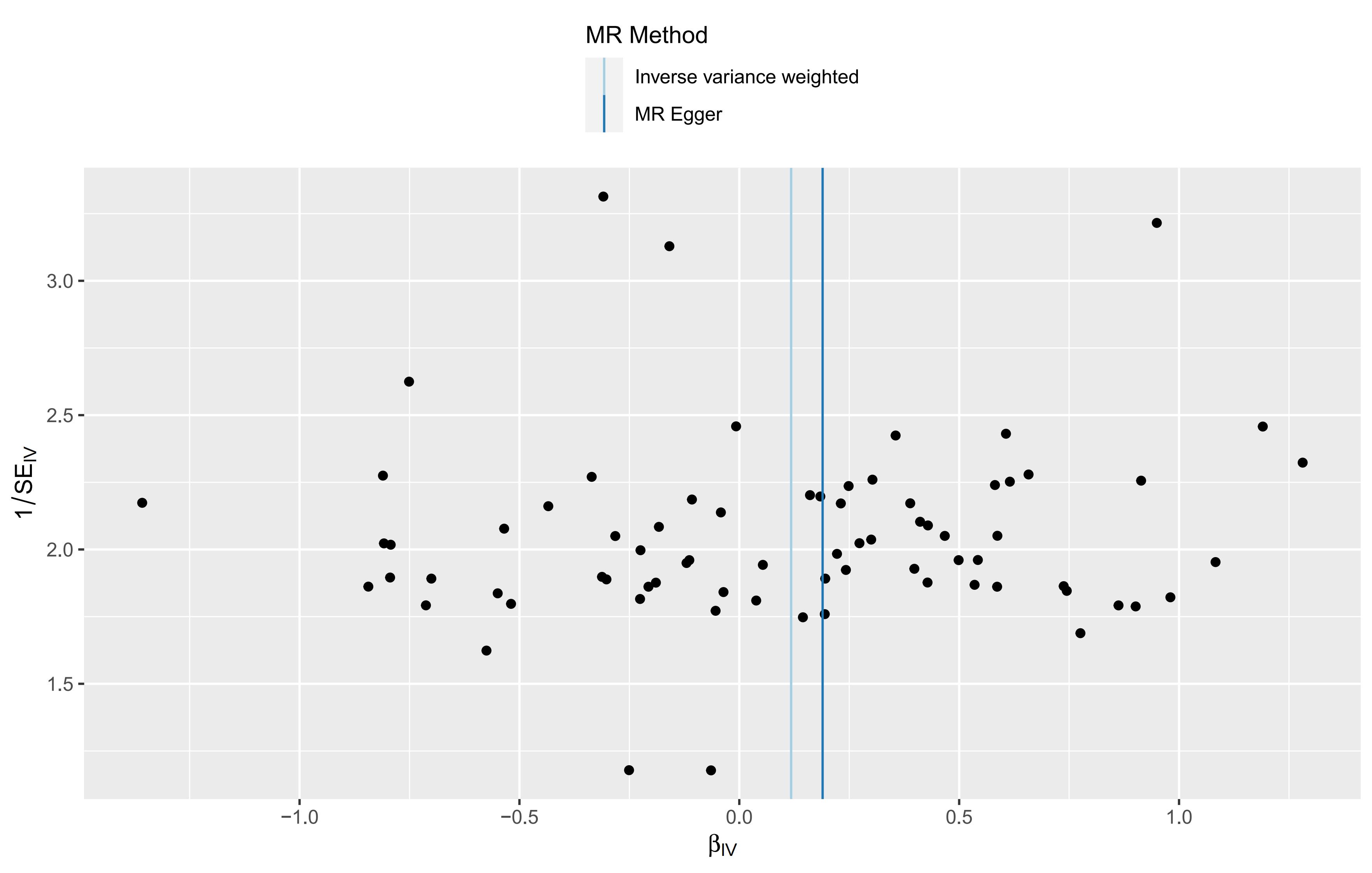

Supplement: Supplementary file 6 [file Datasheet1.docx]
